# Sociodemographic and clinical characteristics in child and youth mental health; comparison of routine outcome measurements of an Australian and Dutch outpatient cohort

**DOI:** 10.1017/S2045796021000652

**Published:** 2021-11-23

**Authors:** S. L. Roest, B. M. Siebelink, H. van Ewijk, R. R. J. M. Vermeiren, C. M. Middeldorp, R. M. van der Lans

**Affiliations:** 1 LUMC-Curium, Centre of Child and Youth Psychiatry, Leiden University, the Netherlands; 2 Youz, Parnassia Group, the Netherlands; 3 Child Health Research Centre, University of Queensland, Australia; 4 Child and Youth Mental Health Service (CYMHS), Children's Health Queensland Hospital and Health Service, Australia

**Keywords:** Child psychiatry, epidemiology, mental health, minority issues and cross-cultural psychiatry, outcome studies

## Abstract

**Aims:**

Although of great value to understand the treatment results for mental health problems obtained in clinical practice, studies using naturalistic data from children and adolescents seeking clinical care because of complex mental health problems are limited. Cross-national comparison of naturalistic outcomes in this population is seldomly done. Although careful consideration is needed, such comparisons are likely to contribute to an open dialogue about cross-national differences and may stimulate service improvement. The aim of this observational study is to investigate clinical characteristics and outcomes in naturalistic cohorts of specialized child and adolescent mental health outpatient care in two different countries.

**Methods:**

Routinely collected data from 2013 to 2018 of 2715 outpatients in the Greater Area of Brisbane, Australia (CYMHS) and 1158 outpatients in Leiden, the Netherlands (LUMC-Curium) were analysed. Demographics, clinical characteristics and severity of problems at start and end of treatment were described, using Children's Global Assessment Scale (CGAS), Health of the Nation Outcome Scales for Children and Adolescents (HoNOSCA) and the parental Strength and Difficulties Questionnaire (SDQ-P).

**Results:**

Routine outcome measures (CGAS, HoNOSCA, SDQ-P) showed moderate to severe mental health problems at start of treatment, which improved significantly over time in both cohorts. Effect sizes ranged between 0.73-0.90 (CYMHS) and 0.57-0.76 (LUMC-Curium). While internalizing problems (mood disorder, anxiety disorder and stress-related disorder) were more prevalent at CYMHS, externalizing developmental problems (ADHD, autism) prevailed at LUMC-Curium. Comorbidity (>1 diagnosis on ICD10/DSM-IV) was relatively similar: 45% at CYMHS and 39 % at LUMC-Curium. In both countries, improvement of functioning was lowest for conduct disorder and highest for somatoform/conversion disorders and obsessive-compulsive disorders (OCD). Overall, 20-40% showed clinically significant improvement (shift from clinical-range at start to a non-clinical-range at the end of treatment), but nearly half of patients still experienced significant symptoms at discharge.

**Conclusions:**

This large-scale outcome study showed both cohorts from Australia and the Netherlands improve during the course of treatment on clinician- and parent-reported measures. Although samples were situated within different contexts and differed in patient profiles, they showed similar trends in improvement per diagnostic group. While 20-40% showed clinically significant change, many patients experienced residual symptoms reflecting increased risk for negative outcome into adulthood. We emphasize cross-national comparison of naturalistic outcomes faces challenges, although it can similarly reveal trends in treatment outcome providing direction for future research: what factors determine discharge from specialized services; and how to improve current treatments in this severely affected population.

## Introduction

Routine outcome measurement (ROM) data offer unique opportunities to study treatment outcomes in clinical practice, and can help to assess the real-world impact of mental health services for children and adolescents (youth). This is illustrated by studies using naturalistic data from specialist child and adolescent mental healthcare services (CAMHS), showing the proportion of patients with reliable improvement, recovery or deterioration (Burgess *et al*., 2015; Wolpert *et al*., 2016), and revealing specific subgroups of patients with greater risk of poor outcome (Garralda *et al*., 2000; Lundh *et al*., 2013; Murphy *et al*., 2015; Edbrooke-Childs *et al*., 2017). Naturalistic data are therefore undeniably necessary in addition to data derived from randomised clinical trials, which often have limited generalisability due to strict selection criteria (Rothwell, 2005; Van Noorden *et al*., 2014).

The availability of ROM-data in different countries enables to compare treatment outcomes on a cross-national level. To date, this has rarely been done. There are opportunities to compare, as countries such as Australia and the Netherlands have implemented the same ROM (CGAS, HoNOSCA and SDQ-P) across CAMHS. Since they also have similar levels of economic development, social and demographic profiles and health outcomes (Prins *et al*., 2011; World Health Organization, 2016; The CommonwealthFund *et al*., 2017), though a different healthcare system, it is interesting to compare mental health treatment outcomes. There have been few Australian reports on children with a broad range of complex problems showing improvement on a range of measures (Brann and Coleman, 2010; Burgess *et al*., 2015; Howe *et al*., 2017; Lu *et al*., 2021). In the Netherlands, large-scale outcome studies in general youth psychiatric outpatient care are surprisingly lacking.

Cross-country comparisons of child psychiatric outcomes are relevant because they can provide insights into how cultural and country-specific system- and individual level-factors impact mental health outcomes (Canino and Alegría, 2008; Ronis *et al*., 2017). Regarding Australia and the Netherlands, country-specific factors that might impact outcomes, include differences in the organisation and financing of CAMHS. In Australia, specialised CAMHS, such as CYMHS, are funded by public health insurance coverage, whereas other mental health care services are often privately funded, so come with sometimes substantial out-of-pocket costs (Callander *et al*., 2017). Due to a high volume of referrals, there is a high threshold of severity and complexity to receive treatment at CYMHS. In contrast, the Dutch government offers free provision of all types of youth mental healthcare, which is regulated at the community level (Hilverdink *et al*., 2015). Based on these system-level factors and our clinical experiences, we would expect a better access to CAMHS in the Netherlands with perhaps lower severity levels of problems in specialised services compared to Australia.

This study described clinical characteristics and outcomes of child psychiatric outpatient treatment in two countries (total sample *n* = 3873), as measured by symptom reduction (HoNOSCA, SDQ-P) and improvement of global functioning (CGAS). Analyses of cohorts focused on a profile of presenting patients, prevalence rates of diagnoses, completion rates and effect sizes of ROM, the proportion of patients with clinical significant change, and change of global functioning among diagnostic groups. Findings are discussed in the light of contextual factors of both organisations. The aim is to open up conversations about the observed similarities and differences in the context of two specialist tertiary CAMHS within Australia and the Netherlands. Thereby this study contributes to the country-specific evidence on the effectiveness of outpatient treatments, and to insights on cross-cultural trends of treatment outcome of youth with complex mental health needs.

## Methods

### Settings

**CYMHS** at Children's Health Queensland Hospital and Health Service, Australia, and **LUMC-Curium**, the Netherlands, are tertiary level specialist services for youth (aged 2–18) with complex and severe mental health problems. They provide community- and hospital-based services in a catchment area comprising 6 00 000 youth across the Greater Brisbane and Pine River regions in Queensland (CYMHS) and 1 62 000 youth in the northern part of the province ‘Zuid-Holland’ (LUMC-Curium). They both work in the presence of – and in collaboration with other mental healthcare providers in the region. Both organisations do not focus on the population with intellectual disabilities, however, comorbid psychiatric symptoms of these patients may be treated. Patient-data and ROM-data (collected at baseline and at case reviews) were registered in online records. In Australia this collection process is regulated in specific guidelines (Burgess *et al*., 2012, 2015; AMHOCN, 2019).

### Subjects

For the current comparison study we followed the same procedure as described by Lu *et al*. (*submitted*) in the selection of patients, with the exception that we focused on a different timeframe, and that we included data from the Eating Disorder-team at CYMHS. These exceptions were made to make selections of CYMHS and LUMC-Curium more comparable.

**Fig. 1.**
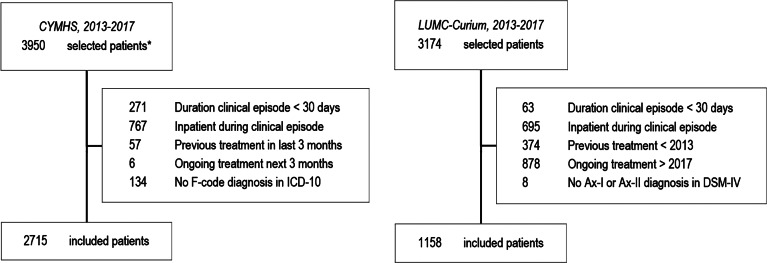
Flow diagram of selected, excluded and included patients for CYMHS and LUMC-Curium

In sum, all patients, aged 5−18 years, attending outpatient mental health services between 2013 and 2018, were selected (Fig. 1). Data related to the first ‘clinical episode’ during the timeframe were described. A ‘clinical episode’ is defined as the total period of care (including all provided services) that is needed for the treatment of a patient across a continuum of care in an integrated system. Clinical episodes may comprise multiple ‘episodes of care’. For example, at CYMHS, a change of setting (or service team) means a new ‘episode of care’ (AMHOCN, 2019). Within LUMC-Curium ‘episodes of care’ were registered following the DBC-structure (*Diagnose-Behandel-Combinatie (Netherlands*)). DBC-registrations span no longer than maximally one year and multiple consecutive DBC's could contribute to a continuous period of care. Therefore, in both samples ‘episodes of care’ have been combined into ‘clinical episodes’ of care for the patient. The start of a clinical episode is defined as ‘admission to CAMHS, without any contact in the previous 3 months’, the end as ‘discharge from CAMHS, without contact in the following 3 months’. To be able to compare outcomes before and after outpatient treatment, we excluded clinical episodes of less than 30 days, youth with inpatient admissions during their clinical episode, and patients without any psychiatric diagnosis (no F-Code in ICD-10 or no Axis I/Axis II diagnosis in DSM-IV) (Fig. 1).

Outpatient care at CYMHS was provided by seven community teams and the Eating Disorder-team, of which 2715 patients were included. At LUMC-Curium outpatient care was provided by several specialised teams, of which 1158 patients were included (Table 1).

**Table 1.** Sociodemographics and comorbidity at intake, across outpatients at CYMHS and LUMC-Curium between 2013 and 2017

	CYMHS *N* = 2715	LUMC-Curium *N* = 1158
Gender, *N* (%)		
Girls	1511 (56)	512 (44)
Boys	1203 (44)	646 (56)
Other	1 (0.0)	–
*Age at intake (year)* Mean (SD)	12.9 (3.2)	11.5 (3.5)
*Ethnic group, N* (%) *		
Australia	2422 (89)	–
New Zealand	88 (3)	–
Other	204 (8)	26 (4)
Netherlands	1 (0.0)	565 (96)
*Aboriginal or Torres Strait Islander Origin* N (%)	296 (7)	–
*Number of clinical episodes,* Mean (SD)	1.1 (0.3)	1.1 (0.2)
Min-Max	1-4	1-2
*Duration of clinical episode (days)*		
Mean (SD)	232 (179.7)	443 (320)
Min-Max		
*Factors influencing mental health Problems related to:*	30-1351	34-1721
− primary support group/upbringing	1685 (62)	625 (54)
− social environment/psychosocial circumstances	692 (26)	283 (24)
− education, literacy	450 (17)	6 (1)
− housing/economic circumstances	89 (3)	18 (2)
− (un)employment	20 (1)	224 (19)
− negative life-event	1104 (39)	–
− other Z-code	579 (21)	–
Family history of mental disorder	1459 (54)	–
Number of psychiatric diagnoses *N* (%)		
1	1482 (55)	715 (62)
2	795 (29)	343 (30)
⩾3	438 (16)	100 (9)
Mean (SD)	1.7 (0.9)	1.5 (0.7)
Min−max	1−6	1−5

*LUMC-Curium: Ethnic group missing *N* = 559.

### Measures

Demographic data included gender, age at start, duration of episode and country of birth. In Australia, ‘indigenous status’, is reported, referring to the first nation people of Australia, i.e., ‘Aboriginal’ or ‘Torres Strait Islander’ (Department of Social Services, 2015). Diagnoses were classified according to the International Statistical Classifications of Diseases 10th revision ICD-10 (WHO, 2016) at CYMHS, or the Diagnostic and Statistical Manual of Mental Disorders (DSM-IV-TR) at LUMC-Curium. Similar diagnoses were combined into major groups (see Appendix A). Psychosocial stressors were registered as Z-codes in ICD-10, or Axis-IV codes in DSM-IV. Diagnostic evaluation (in multidisciplinary meetings) and collection of clinician-rated ROM (CGAS and HoNOSCA) is done by the health care professional responsible for the case. At LUMC-Curium these were well-qualified, certified senior clinician (child- and adolescent psychiatrist or clinical psychologist) with multiple years of clinical experience, at CYMHS also a social worker, psychologist or occupational therapist may have been responsible. Both at CYMHS and LUMC-Curium initial training on HoNOSCA and CGAS was offered to all clinicians. However, completion rates of the training are not available. We assume in both settings sometimes untrained raters completed questionnaires, although the majority of clinicians have been trained.

The CGAS is a clinician-rated measure, assessing the youths’ lowest level of global functioning during previous months, rated on a scale of 1 (lowest functioning) to 100 (excellent functioning) (Shaffer *et al*., 1983). A score below 61 indicates definite pathology, a score below 71 probable pathology (Bird *et al*., 1990; Dyrborg *et al*., 2000). End-of-treatment scores up to 60 are associated with increased risk for negative outcome and adversities in early adulthood (Lundh *et al*., 2016).

The HoNOSCA is a clinician-rated measure, comprising 15 items assessing emotional and behavioural problems in youth during the previous two weeks (Gowers *et al*., 1997, 1999). The first 13 items are used to compute the total score (range 0–52). The last two items address problems with knowledge and understanding and are not reported in most studies. Clinicians score items on a 5-point scale (0–4), ranging from ‘no problems’ to ‘severe problems’, or score ‘9’ if unknown. Ratings ⩾ 2 on any item indicate a clinically significant problem.

The SDQ-P is a parent-rated measure comprising 25 items assessing five domains: emotional problems (EPS), conduct problems (CPS), hyperactivity (HAS), peer problems (PPS) and prosocial behaviour (PSS) (Goodman, 1997). Items are scored on a three-point scale (0–2). The first four domain scores are used to produce a total difficulty score (TDS) ranged 0–40. In Australia, the UK-norms of SDQ-P are used (Woerner *et al*., 2004; Mellor, 2005). According to UK-norms, a TDS-score of 14–16 is categorised as ‘slightly raised’, 17–19 as ‘high’ and ⩾ 20 as ‘very high’ (Goodman, 1997; Goodman and Goodman, 2009). In the Netherlands, the SDQ-P-scores were re-calibrated (Theunissen *et al*., 2016) and cut-off scores vary for age groups: a TDS-score is indicated ‘raised’ if scored ⩾ 15 (aged 4–7) ⩾ 14 (aged 7–12) or ⩾ 12 (aged 13–18).

For the psychometric properties of measures, see Appendix B. Cross-national intraclass correlation coefficient (ICC) was 0.61 for CGAS, and 0.84 for HoNOSCA total score (Hanssen-Bauer *et al*., 2007). All three measures have shown to be valid and useful for international comparison (Woerner *et al*., 2004; Becker *et al*., 2006; Hanssen-Bauer *et al*., 2007), though evidence regarding the SDQ-P is less strong (Stevanovic *et al*., 2017). Because of the limited evidence for cross-cultural validity of SDQ-P (Stevanovic *et al*., 2017), multiple group confirmatory Bi-Factor analysis was applied to assess measurement invariance for the subscale- and total-scale scores at baseline (Rosseel, 2012). In conclusion, while the relative fit indices echoed the results by Stevanovic *et al*., the absolute fit indices provided sufficient evidence supporting descriptive comparisons of Australian and Dutch SDQ-P subscale- and total-scale scores.

### Data analysis

Statistical analyses were conducted using SPSS Version 25 (SPSS Inc., Chicago, IL, USA). A *p*-value <0.05 is regarded as a statistically significant result. The NOCC criteria (AMHOCN, 2019) were used to select valid ROM-ratings. For HoNOSCA, a minimum of 11 of the first 13 items needed a valid rating (score 0–4). Scores of ‘9 – not known’ were regarded as missing, which is suggested in guidelines (Gowers *et al*., 1997; Department of Health and Ageing, 2003). Missing data were excluded from calculations of the total score, which is equivalent as treating them as zero. For SDQ-P, at least three of the five items per domain needed a valid score. Multiple imputation (MI) was used to impute valid, though incomplete SDQ-P-data at CYMHS (3%) and LUMC-Curium (0%).

Statistical analyses concern within-country comparisons between patients' start- and end-ROM-scores. Start- and end-ROM-scores are defined as scores collected within 90 days of the start- or end-of-service date. For all three measures, relevance of improvement was assessed by calculating within-samples effect sizes (Cohen's *d*) using the pooled standard deviation from the start- and end-of-treatment means, taking the correlation between means into account (Morris, 2008). In the group of patients with both start- and end-scores available (*matched scores*), a series of McNemar's tests is used on CGAS, HoNOSCA-items and SDQ-P(TDS), to report on the proportion of patients with clinical significant change. For an individual patient it means the patient shifts from a dysfunctional to a functional population (Wise, 2004). Note that clinical significance for HoNOSCA was analysed on item level, because a criterion for clinical significance for the total score is lacking. Moreover, the reporting of separate items may better reflect important clinical change (Brann and Coleman, 2010; Boon *et al*., 2019). To get an impression of the overall change in symptoms on HoNOSCA, we examined change in the number of clinically significant items between start- and end-of-treatment, using a paired sample *t*-test.

In addition, clinical change was analysed for each diagnostic group. Hereby, we focused on CGAS, because CGAS had most complete data. Per diagnostic group, the proportion of patients scoring in the clinical range was reported for both cohorts. Differences in the proportions between start- and end-of-treatment were tested on significance using a series of McNemar's tests.

## Results

### Patient characteristics and diagnoses

Data show that populations were different considering age and gender distributions (Table 1). Most patients were attending for the first time at CYMHS (92%) and at LUMC-Curium (94%). Internalising problems (mood disorder, anxiety disorder, OCD) were more prevalent at CYMHS; externalising developmental problems (ADHD, autism) prevailed at LUMC-Curium (Table 2). Adjustment disorder, stress-related disorder and eating disorder were also more prevalent at CYMHS. Comorbidity (>1 diagnosis on ICD10 or DSM-IV) was present in 45% at CYMHS and 39% at LUMC-Curium (Table 1).

**Table 2.** All diagnosis registered during the first clinical episode, per patient

	CYMHS			LUMC-Curium		
	All *n* = 2715	Boys *n* = 1203	Girls *n* = 1511	All *n* = 1158	Boys *n* = 646	Girls *n* = 512
	*N* (%)	*N* (%)	*N* (%)	*N* (%)	*N* (%)	*N* (%)
ADHD or ADD	284 (10.5)	211 (17.5)	73 (4.8)	401(34.6)	268 (41.5)	133 (26.0)
Pervasive developmental disorder or autism	227 (8.4)	176 (14.6)	51 (3.4)	268 (23.1)	195 (30.2)	73 (14.3)
Conduct or mixed conduct disorder	192 (7.1)	140 (11.6)	52 (3.4)	72 (6.2)	43 (6.7)	29 (5.7)
Tic disorder	21 (0.8)	20 (1.7)	1 (0.1)	36 (3.1)	31 (4.8)	5 (1.0)
Mood disorder	561 (20.7)	194 (16.1)	367 (24.3)	140 (12.1)	48 (7.4)	92 (18.0)
Anxiety disorder	1058 (39.0)	430 (35.7)	627 (41.5)	175 (15.1)	70 (10.8)	105 (20.5)
Stress-related disorder	430 (15.8)	134 (11.1)	296 (19.6)	33 (2.8)	16 (2.5)	17 (3.3)
Eating disorder	172 (6.3)	19 (1.6)	153 (10.1)	30 (2.6)	6 (0.9)	24 (4.7)
Obsessive−compulsive disorder	84 (3.1)	38 (3.0)	46 (3.0)	18 (1.6)	8 (1.2)	10 (2.0)
Somatoform, conversion or dissociative disorder	35 (1.3)	14 (1.2)	21 (1.4)	62 (5.4)	21 (3.3)	41 (8.0)
Adjustment disorder	409 (15.1)	182 (15.1)	227 (15.0)	8 (0.7)	3 (0.5)	5 (1.0)
Emotional, social and behavioural disorders with onset in childhood/adolescence, incl NOS	364 (13.4)	189 (15.7)	175 (11.6)	156 (13.5)	99 (15.3)	57 (11.1)
Learning/developmental disorder	301 (11.1)	178 (14.8)	123 (8.1)	110 (9.5)	79 (12.2)	31 (6.1)
Intellectual disabilities	47 (1.7)	25 (2.1)	22 (1.5)	52 (4.5)	30 (4.6)	22 (4.3)
Other mental disorders	171 (6.3)	88 (7.3)	83 (5.5)	142 (12.3)	52 (8.0)	90 (17.6)

### Outcomes

#### Cgas

**Fig. 2.**
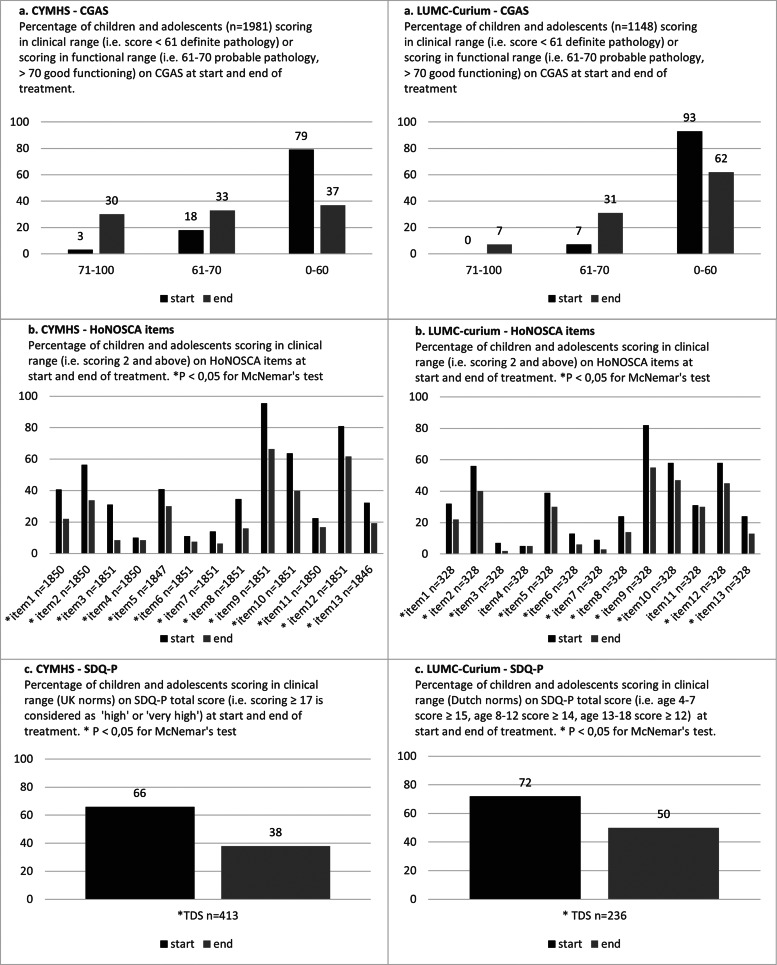
Improvement over time

Table 3 shows moderate initial CGAS ratings in both cohorts, which improved significantly with effect sizes of 0.90 (CYMHS) and 0.76 (LUMC-Curium). Matched start- and end-of-treatment scores were available for 1981 (73%) patients at CYMHS, and 1148 (99%) at LUMC-Curium. Of all patients, 42% at CYMHS and 31% at LUMC-Curium improved from the ‘definitive pathology’ group (score <61) to a group with better functioning (>60), while 27% at CYMHS and 7% at LUMC-Curium reached a functional level (>70) (Fig. 2a). Deterioration of functioning was seen in 14% of patients at CYMHS and 5% at LUMC-Curium. At discharge, still 37% at CYMHS and 62% at LUMC-Curium scored in the clinical range (<61) (Fig. 2a).

**Table 3.** Routine outcome measurements at start and end of treatment

	CYMHS *N* = 2715			LUMC-Curium *N* = 1158		
	Start	End	Effect size*	Start	End	Effect size*
CGAS						
Completion rate, *n* (%)	2395 (88.2)	2136 (78.7)		1158 (100)	1153 (99.6)	
Mean (SD)	53.8 (9.6)	64.9 (12.2)	0.90	52.4 (7.2)	59.2 (8.7)	0.76
HoNOSCA						
Completion rate, *n* (%)	2380 (87.6)	2016 (74.3)		575 (49.7)	523 (45.2)	
Total score, mean (SD)	15.5 (5.8)	10.0 (6.5)	0.84	14.0 (5.7)	10.0 (6.3)	0.57
SDQ-P						
Completion rate, *n* (%)	1949 (71.8)	487 (17.9)		743 (64.2)	296 (25.6)	
TDS, mean (SD)	19.7 (6.9)	14.8 (7.5)	0.73	16.5 (5.7)	12.6 (5.9)	0.61

*Cohen's *d*.

#### HoNOSCA

In total, 8284 HoNOSCA questionnaires at CYMHS and 2911 at LUMC-Curium were completed over time, originating from 2579 (95%) and 905 (78%) patients, respectively. Of these, 2% at CYMHS and 4% at LUMC-Curium were deemed invalid, due to missing data. Mean total scores improved significantly in both cohorts with effect sizes of 0.84 (CYMHS) and 0.57 (LUMC-Curium) (Table 3).

Matched start- and end-of-treatment scores were available for 1851 (68%) patients at CYMHS, and for 321 (28%) patients at LUMC-Curium. The mean number of clinically significant items per patient decreased from 5.3 (SD 2.0) at start to 3.4 (SD 2.5) at end-of-treatment at CYMHS (*p* = 0.000), and from 4.4 (SD 2.0) to 3.1 (SD 2.3) at LUMC-Curium (*p* = 0.000). A list of the HoNOSCA-items is found in Appendix C. At discharge, significantly less patients scored in the clinical range on all of the 13 HoNOSCA-items at CYMHS. At LUMC-Curium, also all items showed a significant difference, except items 4 and 11 (Fig. 2b). Besides the statistical significant difference on item-level, which might be influenced by the different sample sizes of cohorts, we looked at trends that were visible in both cohorts. At CYMHS and at LUMC-Curium the most common problem faced by presenting outpatients regarded emotional and related symptoms (item 9), which was reported by 95% (CYMHS) and 82% (LUMC-Curium) of patients. Other prevalent symptoms included relational problems with peers (item 10) or family (item 12); this was reported by 64 and 81%, respectively, at CYMHS, and 58 and 58%, respectively, at LUMC-Curium. Although for a significant proportion of patients these issues resolved after treatment, they remained the most frequently reported problems upon discharge. A substantial difference between cohorts was the initial scoring on problems with self-injury (item 3), which was 31% at CYMHS compared to 7% at LUMC-Curium.

#### SDQ-P

In total, 3380 SDQ-P at CYMHS and 1198 at LUMC-Curium were completed over time, originating from 2067 (76%) patients and 499 (43%) patients, respectively. Parental completion rates (SDQ-P) were lower compared to the clinician-rated measurements (Table 3). Patients significantly improved in both cohorts, with effect sizes of 0.73 at CYMHS and 0.61 at LUMC-Curium. Matched start- and end-of-treatment scores were available for 413 (15%) patients at CYMHS, and 236 (20%) at LUMC-Curium. Twenty-nine percent of patients at CYMHS and 23% at LUMC-Curium, improved from a clinical TDS-score at start, to a non-clinical TDS-score at end of treatment (Fig. 2c). However, according to parents, still 38% at CYMHS, and 50% at LUMC-Curium experienced significant problems at discharge. Hence, in both countries the parent ratings on the SDQ-P corroborated the clinician ratings on CGAS and HoNOSCA.

#### Outcome per diagnostic group (Table 4)

Exploration concentrated on the proportions of patients showing clinical relevant change on CGAS. At CYMHS and at LUMC-Curium, the largest ΔCGAS was seen in patients with somatoform, conversion or dissociative disorders and OCD.^1^ The lowest ΔCGAS was seen in patients with conduct disorder. In both samples, the groups of intellectual disabilities and conduct disorder had highest proportion of patients (70–80%) scoring in the clinical range (<61) at end of treatment. In the groups of mood disorders and anxiety disorders a higher proportion of patients showed clinical relevant change (40–50%) compared to the groups of ASD, ADHD or ‘Emotional, social and behavioural disorders, childhood/adolescence onset’ (25–35%). Again, these trends were seen in both cohorts.

**Table 4.** Outcomes per diagnostic group, ordered by ΔCGAS

	CYMHS *N* = 2715						LUMC-Curium *N* = 1158					
	*n*	CGAS Start	ΔCGAS	% clinical range start Score <61	% clinical range end Score <61	% Clinically improved*	*n*	CGAS Start	ΔCGAS	% clinical range start Score <61	% clinical range end Score <61	% Clinically improved*
All	1981	53.8 (9.6)	11.1	79	37	42**	1148	52.4 (7.2)	6.8	93	63	31**
Somatoform, conversion or dissociative disorder	25	51.3 (15.7)	17.6	72	24	48**	60	49.8 (11.1)	12.8	95	45	50**
OCD	66	51.1 (9.7)	15.3	83	27	56**	18	53.3 (5.7)	12.9	89	33	56**
Tic disorder	18	52.4 (11.8)	14.3	89	33	56**	35	54.8 (6.7)	6.7	83	46	37**
Mood disorder	415	53.0 (8.9)	13.2	85	32	53**	138	48.6 (8.6)	10.7	98	54	44**
Anxiety disorder	794	53.2 (9.2)	12.3	81	35	46**	173	51.6 (8.0)	10.3	96	54	42**
Eating disorder	138	53.3 (9.7)	12.0	77	39	38**	30	48.6 (10.9)	10.0	97	57	40**
Learning /developmental disorder	229	50.8 (9.3)	11.2	87	47	40**	110	52.9 (5.7)	6.3	94	61	33**
Adjustment disorder	292	56.6 (9.4)	10.8	71	28	42**	8	54.8 (2.8)	13.7	100	25	75**
ASD	159	50.5 (9.6)	10.4	87	52	35**	267	51.1 (6.2)	5.8	97	71	26**
Stress-related disorder	323	53.7 (9.6)	10.2	81	41	39**	33	47.9 (4.8)	7.6	100	73	27**
ADHD or ADD	223	52.6 (9.5)	10.0	83	48	35**	399	53.3 (5.2)	5.6	94	67	27**
Mental retardation	31	46.7 (9.2)	9.5	97	71	26**	52	49.4 (6.7)	5.4	98	81	17**
Other disorder	131	52.9 (9.8)	9.1	86	47	40**	140	52.5 (8.6)	6.0	88	64	24**
Emotional, social and behavioural disorders with onset in childhood /adolescence, incl NOS	263	53.7 (9.7)	9	80	45	35**	156	52.7 (5.8)	5.9	93	67	26**
Conduct or mixed conduct disorder	123	53.3 (8.7)	6.3	83	59	24**	72	50.7 (6.2)	4.6	96	83	13**

* Proportion of patients who shifts from a dysfunctional (score <61) to a functional population (score > 60).

** *P* < 0.05 for McNemar's test.

## Discussion

This study investigated clinical characteristics and treatment outcomes of child psychiatric outpatient care in Australia and the Netherlands. The results reveal both organisations differed in patient profiles and prevalence rates of diagnostic groups. However, similar trends were found. In both countries, patients presented with moderate-to-severe problems and showed significant improvement over time. Improvement was clinically relevant in 20–40% of patients, dependent on diagnostic group, and reported by both clinician (CGAS, HoNOSCA) and parents (SDQ-P). A significant proportion of patients experienced residual symptoms.

The similarities observed between the countries and organisations align with outcomes in prior studies of outpatients with severe and complex mental health problems. First, severity of problems at start-of-treatment was high in both cohorts (79–93 and 66–72% in clinical range on CGAS and SDQ-P, respectively), reflecting the specialised settings, similar to other specialised CAMHS (Garralda *et al*., 2000; Becker *et al*., 2006; Lundh *et al*., 2013; Howe *et al*., 2017; Vugteveen *et al*., 2018). Second, the treatment outcome between 20 and 40% improving from dysfunctional to a functional level of symptoms were comparable to other studies in this population, in which the percentages range from 20 to 50% (Brann and Coleman, 2010; Lundh *et al*., 2016; Wolpert *et al*., 2016; Howe *et al*., 2017). This is in line with the impression that response to treatments varies little across cultures (Canino and Alegría, 2008). Third, gender patterns of psychiatric disorders were similar in both samples and consistent with findings from epidemiology studies, reporting girls have increased risks for mood- and other internalising disorders with increasing age, and boys are more often diagnosed with ADHD and conduct disorders (Cohen *et al*., 1993; Garland *et al*., 2001; Patel *et al*., 2007). Fourth, patients with conduct disorder improved less after treatment and together with the group with intellectual disabilities (treated for their comorbid psychiatric symptoms) they showed most residual symptoms compared to other diagnostic groups. In addition, in both samples, lower proportions of patients with clinical relevant change were found for externalising developmental problems (ADHD, autism) compared to internalising disorders. These trends might be explained by the more chronic and persistent course of externalising disorders (Wittchen *et al*., 2000; Roy *et al*., 2016; Ormel *et al*., 2017). Nevertheless, this may draw our attention to the specific subgroups in risk of poorer outcomes, to optimise their treatments and to make their problems as manageable as possible. The finding is in line with previous studies in which children with autism and mental retardation seemed more at risk for poor outcome (Lundh *et al*., 2013; Edbrooke-Childs *et al*., 2017).

In contrast to our expectations, both cohorts showed similar levels of severity at start. Based on the differences in accessibility of the service, we expected that youth attending CYMHS would have higher initial scores. Furthermore, a substantial part of patients experienced significant problems at the end of treatment: 37% at CYMHS and 50–60% at LUMC-Curium. The following factors could play a role in both countries. In the Netherlands, the government has decentralised and transformed the youth care system since 2014, aiming to put more effort in prevention, and to reduce the use of specialised care (Bosscher, 2014; Hilverdink *et al*., 2015). Consequently, specialised settings are pressured to refer to universal services if possible, in order to reduce treatment time and costs. In Australia, the high inflow of acute cases in specialised settings is putting pressure on the outflow of patients (Lu *et al*., 2021). Once symptoms have improved, patients are possibly referred to other services in Australia, which often require out-of-pocket costs. Although we can only speculate, these organisational factors in both countries might have contributed to the discharge (or transition) of patients with subclinical or residual symptoms. However, transitioning of services may increase the risk for drop-out, and clinical scores at discharge reflect risk for persistence of symptoms into adulthood (Lundh *et al*., 2016). We recognise that it may not be realistic to treat all patients until scores are in the non-clinical range, as some youth will experience enduring and chronic problems. Nevertheless, recovery should be the aim of treatment and should not be hindered by the financial climate.

In the presence of similar trends, both cohorts show different patient profiles which can possibly be explained by contextual factors of mental health organisation. First, in Australia treatment of youth with ADHD or autism is for the most part done by general or developmental paediatricians, while in the Netherlands the far majority of these patients are seen by child and adolescents psychiatrists. As a result, the percentage of youth with ADHD or autism was three times lower at CYMHS than at LUMC-Curium, and youth with internalising problems prevailed. Consequently, at CYMHS the proportion of boys is lower and the mean age at intake is higher. At LUMC-Curium the prevalence rate of eating disorders, adjustment disorders and trauma- and stress-related disorders were lower than at CYMHS. For eating disorders, this difference is possibly explained by the presence of another CAMHS-organisation specialised in eating disorders in the same region as LUMC-Curium. For adjustment disorder, it is likely due to a difference in insurance coverage, where treatment for this disorder is covered in Australia, but not covered in the Netherlands. Regarding stress-related disorders, the difference might be related to the ‘acute stress disorder’, which is regularly diagnosed at CYMHS, but is lacking at LUMC-Curium. This probably also is due to financial issues in the Netherlands. Second, the lower scores on SDQ-P at LUMC-Curium were expected because population-based studies showed lower norm-scores for the Dutch population (Theunissen *et al*., 2016), compared to the Australian population in which the spread of scores is in line with the UK norms (Mellor, 2005). At last, as the difference in duration of treatment between samples might represent an actual difference, we cannot exclude the contribution of methodological factors such as different methods of registration, more regular evaluations at CYMHS, or other system-level or individual-level factors.

Overall these findings point to the importance of including contextual factors, such as organisation of healthcare providers, the geographic availability of services, insurance coverage and methods of registration, as a background when interpreting results. The challenge for future research is to incorporate these factors in their models.

Although studying clinical naturalistic data carries undeniable relevance, this study bears some noteworthy limitations. The high degree of missing data and the lower level of completion rates of ROM (especially for the SDQ-P) makes data possibly biased (Wolpert and Rutter, 2018). More effort is needed in collecting higher completion rates, to achieve better generalisability. Further, in both naturalistic settings, no data were available on interrater-reliability within these organisations. Nevertheless, when carefully interpreting these data, the use in the context of complex adaptive mental health systems, can support dialogue on service improvement and learning (Wolpert and Rutter, 2018). Furthermore, results in change of ROM should be interpreted with care, because changes could have occurred naturally or may be due to factors as regression to the mean or other environmental factors influencing a child's outcome. In addition, access to ‘start’ CGAS ratings might have biased the clinician, when rating the ‘end’ CGAS. However, differences in CGAS change were seen among diagnostic groups, which moreover emerged in both countries. This suggests clinicians involved nuanced ratings, rather than simply rated patients as improved. Furthermore, findings were corroborated by a parent-report measure, which lessened the risk of clinician bias. Unfortunately, we were not able to include data on the type of intervention, although it is a factor associated with outcome. It will be of interest for future studies, to look into differences in treatment provision related to outcome for specific populations. Unfortunately, the data about number of visits, or number of direct time (treatment minutes) turned out to be incomparable. Future studies conducted with data from clinical practice are advised to pay more attention to collecting data about the intensity of treatment in a standardised way, so that data can be better compared. In sum, these data have great potential to broaden the understanding of complex and severe mental health problems in children and adolescents. The present observational study revealed substantial improvement in both cohorts of CYMHS (AUS) and LUMC-Curium (NL) and similar trends in outcome across countries. However, many patients experienced residual symptoms at discharge, which increases the risk for impairment of functioning into adulthood. These data guide future research to further investigate what factors influence discharge from specialised services, and how to improve current treatments in this severely affected population.

## Footnotes

1 At LUMC-Curium, patients with adjustment disorder show the highest ΔCGAS. This is not reported, because of the small group size (*n*=8).

## Supplementary material

For supplementary material accompanying this paper visit https://doi.org/10.1017/S2045796021000652. click here to view supplementary material

## Availability of data and materials

Data of this study and are available upon request.
