# Sociodemographic and clinical characteristics in child and youth mental health; comparison of routine outcome measurements of an Australian and Dutch outpatient cohort – ADDENDUM

**DOI:** 10.1017/S2045796022000221

**Published:** 2022-06-01

**Authors:** S. L. Roest, B. M. Siebelink, H. van Ewijk, R. R. J. M. Vermeiren, C. M. Middeldorp, R. M. van der Lans

**Affiliations:** 1 LUMC-Curium, Centre of Child and Youth Psychiatry, Leiden University, the Netherlands; 2 Youz, Parnassia Group, the Netherlands; 3 Child Health Research Centre, University of Queensland, Australia; 4 Child and Youth Mental Health Service (CYMHS), Children's Health Queensland Hospital and Health Service, Australia

An abstract was missing from the above article. This has since been updated in the online PDF and HTML versions. The authors and Publisher apologise for the error.
