# Supplementary material for: Sociodemographic and clinical characteristics in child and youth mental health; comparison of routine outcome measurements of an Australian and Dutch outpatient cohort
Source: Epidemiol Psychiatr Sci. 2021 Nov 23;30:e74. doi: 10.1017/S2045796021000652 (PMC8611930; doi:10.1017/S2045796021000652)
Supplement: Supplementary file 1 [file S2045796021000652sup001.docx]

APPENDICES

Appendix A

Appendix B

Appendix C

| **Table A1. Major diagnostic groups** | |  |
| --- | --- | --- |
| Diagnostic group | ICD-10* | DSM-IV-TR |
| ADHD or ADD | Hyperkinetic disorders (F90.0-F90.9) - Disturbance of activity and attention - Hyperkinetic conduct disorder - Other hyperkinetic disorders / unspecified | Attention deficit Hyperactivity disorder (314)  - Attention deficit disorder with hyperactivity, combined type - attention deficit disorder, predominant inattention type - attention deficit disorder NOS |
| Pervasive developmental disorder or Autism | Pervasive developmental disorders (F84.0-F84.9) - Childhood autism,  - atypical autism  - Rett syndrome - other childhood disintegrative disorder - Asperger Syndrome - other pervasive developmental disorder / unspecified | Pervasive developmental disorders (299)  - Autistic disorder  - Rett’s disorder - Childhood Disintegrative Disorder - Asperger syndrome  - Pervasive developmental disorder NOS |
| Tic disorder | Tic disorders (F95.0-F95.9) - Combined vocal and multiple motor tic disorder [de la Tourette] **-** Transient tic disorder - Chronic motor or vocal tic disorder  - Other tic disorders / unspecified | Tic disorders (307.2)  - Gilles de la Tourette syndrome - transient tic disorder  - Chronic motor or vocal tic disorder  - Tic disorder NOS |
| Conduct or mixed conduct disorder | Diagnosis of conduct disorder or mixed conduct disorder (F91-F92)  - Conduct disorder confined to the family context  - Unsocialized conduct disorder  - Socialized conduct disorder  - Oppositional defiant disorder  - Other conduct disorders/ unspecified | Conduct disorders (312 - 313) - Conduct disorder, (childhood, adolescent, unspecified onset) - Disruptive behavior disorder NOS  - Oppositional defiant disorder  - Impulse control disorder NOS  - intermittent explosive disorder |
| Mood disorder | Mood [affective] disorders (F30-F39) - depressive episode - recurrent depressive disorder - persistent mood (affective) disorders - Manic episode - Bipolar affective disorder  - other mood (affective) disorders / unspecified | Mood disorders (296, 300.4, 301.1) - Dysthymic disorder  - Major depressive disorder, (single episode / recurrent episode)  - Depressive disorder NOS - Bipolar I disorder, Bipolar II disorder - Cyclothymic disorder - Mood disorder NOS |
| Anxiety disorder | Anxiety disorders (F40, F41, F93)  - Generalised Anxiety disorder  - Panic disorder  - Phobic anxiety disorders  - Other anxiety disorders / anxiety disorder unspecified  - Separation anxiety disorder | Anxiety disorder (300.0, 300.2) - Generalized anxiety disorder - Panic disorder (with / without agoraphobia)  - Specific Phobia / Social Phobia - Anxiety disorder NOS - Separation anxiety disorder |
| Stress-related disorder | Reactions to severe stress (F43.0,F43.1,F43.8,F43.9) - Acute stress reaction  - Post-traumatic stress disorder  - Other reactions to severe stress / unspecified | Stress disorders (309.8, 308.3)  - Post-traumatic stress disorder  - Acute stress disorder |
| Eating disorder | Eating disorders (F50) - Anorexia Nervosa - Bulimia Nervosa - Other eating disorder / Eating disorder unspecified | Eating disorders (307.1, 307.5)  Anorexia nervosa Bulimia Nervosa  Eating disorder NOS |
| Obsessive-compulsive disorder | Obsessive-compulsive disorder (F42)  - predominantly obsessive thoughts - predominantly compulsive acts - mixed obsessional thoughts and acts - Obsessive-compulsive disorder, unspecified | Obsessive-compulsive disorder (300.3) |
| Somatoform, conversion and dissociative disorder | Somatoform, conversion and dissociative disorders (F44-F45) - Somatization disorder  - Undifferentiated somatoform disorder  - hypochondriacal disorder - somatoform autonomic dysfunction - Persistent somatoform pain disorder - Other somatoform disorder / unspecified - Dissociative amnesia / fugue / stupor - Dissociative motor disorder / convulsion/ anaesthesia and sensory loss - Trance and possession disorder - Mixed dissociative (conversion) disorder - Other dissociative (conversion) disorder / unspecified | Somatoform disorders (300.1, 300.8) - Somatization disorder  - Undifferentiated somatoform disorder  - Conversion disorder - Pain disorder - Hypochondriasis - Body dysmorphic disorder - Somatoform disorder NOS - Depersonalization disorder - Dissociative amnesia  - Dissociative identity disorder - Dissociative disorder NOS |
| Adjustment disorder | Adjustment disorders (F43.2) | Adjustment disorders (309) - adjustment disorder with depressed mood  - adjustment disorder with anxiety  - adjustment disorder with mixed anxiety and depressed mood - adjustment disorder with disturbance of conduct - adjustment disorder with mixed disturbance of emotions and conduct  - adjustment disorder unspecified |
| Emotional, social and behavioural disorders with onset in childhood /adolescence, incl NOS | Emotions, behavioral or social functioning disorders with onset specific to childhood or adolescence (F94, F98, F93.3)  - Elective mutism - reactive attachment disorder of childhood - disinhibited attachment disorder of childhood - other childhood disorder social functioning / unspecified - sibling rivalry disorder - other emotional childhood disorder / unspecified  - Nonorganic enuresis / encopresis  - Feeding disorder of infancy and childhood  - Pica of infancy and childhood  - Stereotyped movement disorders  - Stuttering [stammering]  - Cluttering  - Other specified behavioural and emotional disorders with onset usually occurring in childhood and adolescence / unspecified | Disorders of infancy, childhood or adolescence (313.2, 313.8, 313.9, 307)  - Elective mutism - reactive attachment disorder of childhood - disinhibited attachment disorder of childhood  - Nonorganic enuresis / encopresis  - Feeding disorder of infancy or early childhood  - Pica of infancy and childhood  - Rumination disorder  - Stereotypic movement disorder  - Stuttering [stammering]  - Communication disorder NOS  - Disorder of infancy, childhood or adolescence NOS |
| Learning / developmental disorder | Other specific developmental disorders (F80-F83, F88, F89) - Specific speech articulation disorder  - Expressive language disorder - Receptive language disorder - Acquired aphasia with epilepsy [Landau-Kleffner] - Other developmental disorders of speech and language / unspecified - Specific reading disorder  - Specific spelling disorder  - Specific disorder of arithmetical skills - Mixed disorder of scholastic skills  - Other developmental disorders of scholastic skills / unspecified - Specific developmental disorder of motor function - Mixed specific developmental disorders  - Other disorders of psychological development / unspecified | Learning disorders (315) - Reading disorder - Mathematics disorder - Disorder of written expression  - Developmental coordination disorder - Expressive language disorder - Mixed receptive-expressive language disorder  - Phonological disorder  - Learning disorder NOS |
| Intellectual disabilities | Mental retardation (F70 - F79)  - Mild mental retardation  - Moderate mental retardation  - Severe mental retardation  - profound mental retardation  - other mental retardation / unspecified | Mental retardation (317-319)  - Mild mental retardation  - Moderate mental retardation  - Severe mental retardation - profound mental retardation  - Mental retardation; severity unspecified |
| Other mental disorder | (F02-09, F10-19, F20-29, F51, F52, F54-55 F59-63, F64, F66, F68) - Gender Identity disorders  - sexual dysfunction  - Nonorganic sleep disorders  - Mental and behavioral disorders due to psychoactive substance use  - Specific or mixed personality disorders  - other disorders of personality and behaviour  - Schizophrenia, schizotypal and delusional disorders  - Organic mental disorders - psychological or behavioural disorders associated with sexual development and orientation - Habit and impulse disorders (Trichotillomania, pyromania, kleptomania)  - psychological and behavioural factors associated with disorders or diseases classified elsewhere  - unspecified mental disorder | Other mental disorders (291, 292, 295, 297, 298.8-9, 300.9, 301, 302.6-302.8, 307.4, 305, 313.8, 995) - Gender identity disorder (children / adolescents) - Gender identity disorder NOS - sexual dysfunction - Identity problem - Schizofrenia and other psychotic disorders - Primary insomnia / hypersomnia / Insomnia related to.. (Ax I or AxII) - Parasomnias, other sleep disorders - Impulse control disorders not elsewhere classified (Trichotillomania, pyromania, kleptomania) - substance use related disorders (alcohol, cannabis, amphetamine, NOS)  - personality disorders (Cluster A, B, C or NOS) - Physical or sexual abuse of child - psychological factors affecting a medical condition - Mental disorder due to…. (somatic condition) - unspecified mental disorder |
| *https://icd.who.int/browse10/2015/en#/V | | |

Appendix B

| **Table B1. Psychometric properties of used measures** | |
| --- | --- |
| CGAS | The psychometric properties show moderate reliability and validity, even when used in large clinical settings with (sometimes untrained) raters from different professions (Hanssen-Bauer *et al.*, 2007a; Lundh *et al.*, 2010). Intra class correlations (ICC) in clinical settings ranged from 0.59 to 0.63 (Rey *et al.*, 1995; Dyrborg *et al.*, 2000; Hanssen-Bauer *et al.*, 2007a) and was 0.73 for untrained raters (Lundh *et al.*, 2010). Cross-national ICC was found to be 0.61 (Hanssen-Bauer *et al.*, 2007b).  The CGAS can detect changes due to therapeutic interventions (Steinhausen, 1987). |
| HoNOSCA | Reports so far show HoNOSCA has good interrater reliability and face validity (Gowers *et al.*, 1999; Brann *et al.*, 2001; Bilenberg, 2003; Hanssen-Bauer *et al.*, 2007a) and appears to be sensitive to change (Garralda *et al.*, 2000; Brann *et al.*, 2001; Bilenberg, 2003; Brann & Coleman, 2010; Iachina & Bilenberg, 2012). ICC for HoNOSCA total score, measured in clinical settings, appears moderate (0.52-0.72) (Brann *et al.*, 2001) to substantial (0.81) (Hanssen-Bauer *et al.*, 2007a). Cross-national ICC was found to be 0.84 for HoNOSCA total score (Hanssen-Bauer *et al.*, 2007b).  For HoNOSCA, normative data from a non-clinical population are lacking. |
| SDQ-P | The internal consistency has been reported as 0.82 and the SDQ-P can detect changes due to therapeutic interventions (Goodman, 1997, 2001; Muris *et al.*, 2003). |

References appendix B

**Bilenberg, N.** (2003). Health of the Nation Outcome Scales for Children and Adolescents (HoNOSCA): Results of a Danish field trial. *European Child and Adolescent Psychiatry*, **12**(6), 298–302.

**Brann, P., Coleman, G.** (2010). On the Meaning of Change in a Clinician’s Routine Measure of Outcome: HoNOSCA. *Australian & New Zealand Journal of Psychiatry*, **44**(12), 1097–1104.

**Brann, P., Coleman, G., Luk, E.** (2001). Routine Outcome Measurement in a Child and Adolescent Mental Health Service: An Evaluation of HoNOSCA. *Australian & New Zealand Journal of Psychiatry*, **35**(3), 370–376.

**Dyrborg, J., Warborg Larsen, F., Nielsen, S., Byman, J., Buhl Nielsen, B., Gautrè-Delay, F.** (2000). The Children’s Global Assessment Scale (CGAS) and Global Assessment of Psychosocial Disability (GAPD) in clinical practice - Substance and reliability as judged by intraclass correlations. *European Child and Adolescent Psychiatry*, **9**(3), 195–201.

**Garralda, M. E., Yates, P., Higginson, I.** (2000). Child and adolescent mental health service use. HoNOSCA as an outcome measure. *British Journal of Psychiatry*, **177**(JUL.), 52–58.

**Goodman, R.** (1997). The strengths and difficulties questionnaire: A research note. *Journal of Child Psychology and Psychiatry and Allied Disciplines*, **38**(5), 581–586.

**Goodman, R.** (2001). Psychometric properties of the strengths and difficulties questionnaire. *Journal of the American Academy of Child and Adolescent Psychiatry*, **40**(11), 1337–1345.

**Gowers, S. G., Harrington, R. C., Whitton, A., Lelliott, P., Beevor, A., Wing, J., Jezzard, R.** (1999). Brief scale for measuring the outcomes of emotional and behavioural disorders in children. *British Journal of Psychiatry*, **174**(5), 413–416.

**Hanssen-Bauer, K., Aalen, O. O., Ruud, T., Heyerdahl, S.** (2007a). Inter-rater reliability of clinician-rated outcome measures in child and adolescent mental health services. *Administration and Policy in Mental Health and Mental Health Services Research*, **34**(6), 504–512.

**Hanssen-Bauer, K., Gowers, S., Aalen, O. O., Bilenberg, N., Brann, P., Garralda, E., Merry, S., Heyerdahl, S.** (2007b). Cross-national reliability of clinician-rated outcome measures in child and adolescent mental health services. *Administration and Policy in Mental Health and Mental Health Services Research*, **34**(6), 513–518.

**Iachina, M., Bilenberg, N.** (2012). Measuring reliable change of emotional and behavioural problems in children. *Psychiatry Research*, **200**(2–3), 867–871.

**Lundh, A., Kowalski, J., Sundberg, C. J., Gumpert, C., Landén, M.** (2010). Children’s Global Assessment Scale (CGAS) in a naturalistic clinical setting: Inter-rater reliability and comparison with expert ratings. *Psychiatry Research*, **177**(1–2), 206–210.

**Muris, P., Meesters, C., Van den Berg, F.** (2003). The Strengths and Difficulties Questionnaire (SDQ) further evidence for its reliability and validity in a community sample of Dutch children and adolescents. *European Child and Adolescent Psychiatry*, **12**(1), 1–8.

**Rey, J. M., Starling, J., Wever, C., Dossetor, D. R., Plapp, J. M.** (1995). Inter‐Rater Reliability of Global Assessment of Functioning in a Clinical Setting. *Journal of Child Psychology and Psychiatry*, **36**(5), 787–792.

**Steinhausen, H.-C.** (1987). Global Assessment of Child Psychopathology. *Journal of the American Academy of Child & Adolescent Psychiatry*, **26**(2), 203–206.

Appendix C

| **Table C1. Description of the HoNOSCA items** | |
| --- | --- |
| HoNOSCA | description |
| Item 1 | Problems with disruptive, antisocial or aggressive behaviour |
| Item 2 | Problems with over-activity, attention or concentration |
| Item 3 | Non-accidental self-injury |
| Item 4 | Problems with alcohol, substance or solvent misuse |
| Item 5 | Problems with scholastic or language skills |
| Item 6 | Physical illness or disability problems |
| Item 7 | Problems associated with hallucinations delusions or abnormal perceptions |
| Item 8 | Problems with non-organic somatic symptoms |
| Item 9 | Problems with emotional and related symptoms |
| Item 10 | Problems with peer relationships |
| Item 11 | Problems with self-care and independence |
| Item 12 | Problems with family life and relationships |
| Item 13 | Poor school attendance |
